# Supplementary material for: Disruption of the FGFR1-FGF23-Phosphate Axis and Targeted Therapy in a Murine Model of Osteoglophonic Dysplasia
Source: bioRxiv. 2025 Nov 14:2025.11.14.680268. Preprint. [Version 1] doi: 10.1101/2025.11.14.680268 (PMC12747257; doi:10.1101/2025.11.14.680268)
Supplement: Supplement 1 [file media-1.pdf]

Disruption of the FGFR1-FGF23-Phosphate Axis and Targeted Therapy in a Murine Model of Osteoglophonic Dysplasia

Supplemental material:

Supplemental Figure S1 to Supplemental Figure S3;

Supplemental Table ST1, Supplemental Table ST2

Supplemental Data SD1

Supplemental Figure S1

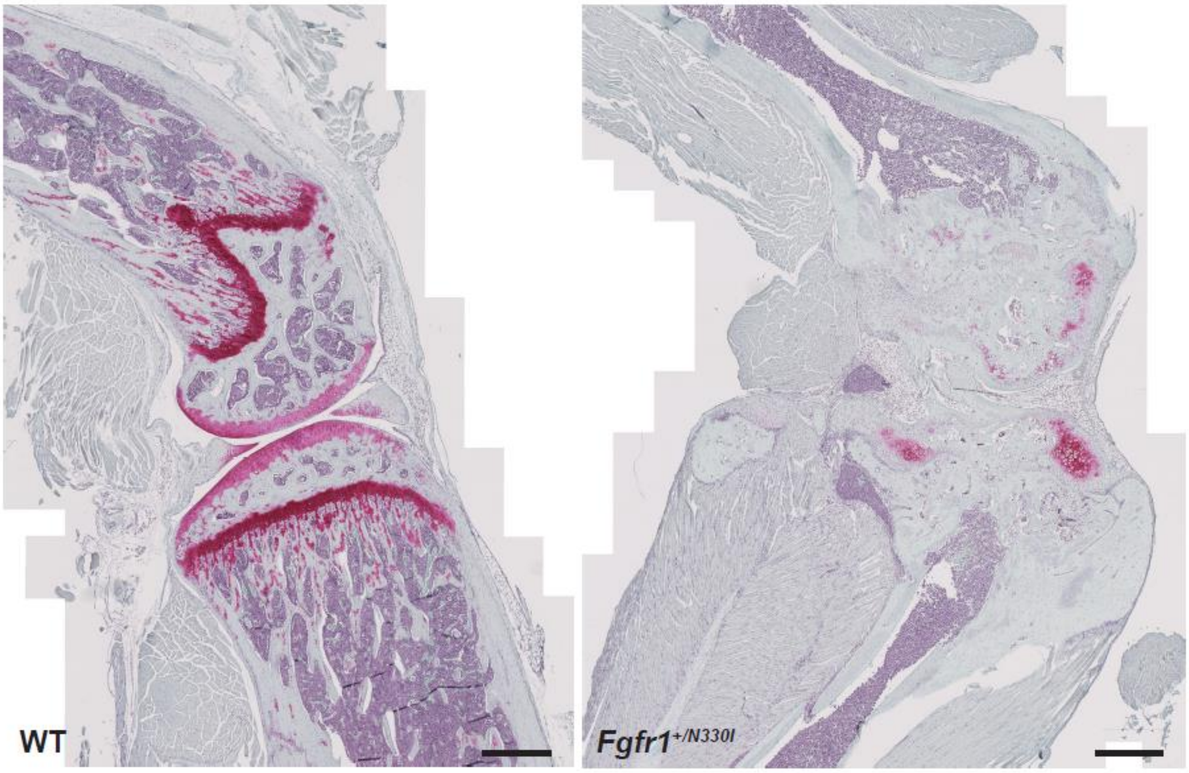

Supplemental Figure S1. ***Fgfr1**$^{+/N330I}$* **mice display disorganization of cartilage extracellular matrix at 12 weeks.**

Safranin O/Fast green (SafO/FG) staining for proteoglycans showed disorganization of cartilage in 12-week-old *Fgfr1*$^{+/N330I}$ mice as indicated by the impaired distribution of the extracellular matrix (red). Scale bar = 500 µm.

Supplemental Figure S2

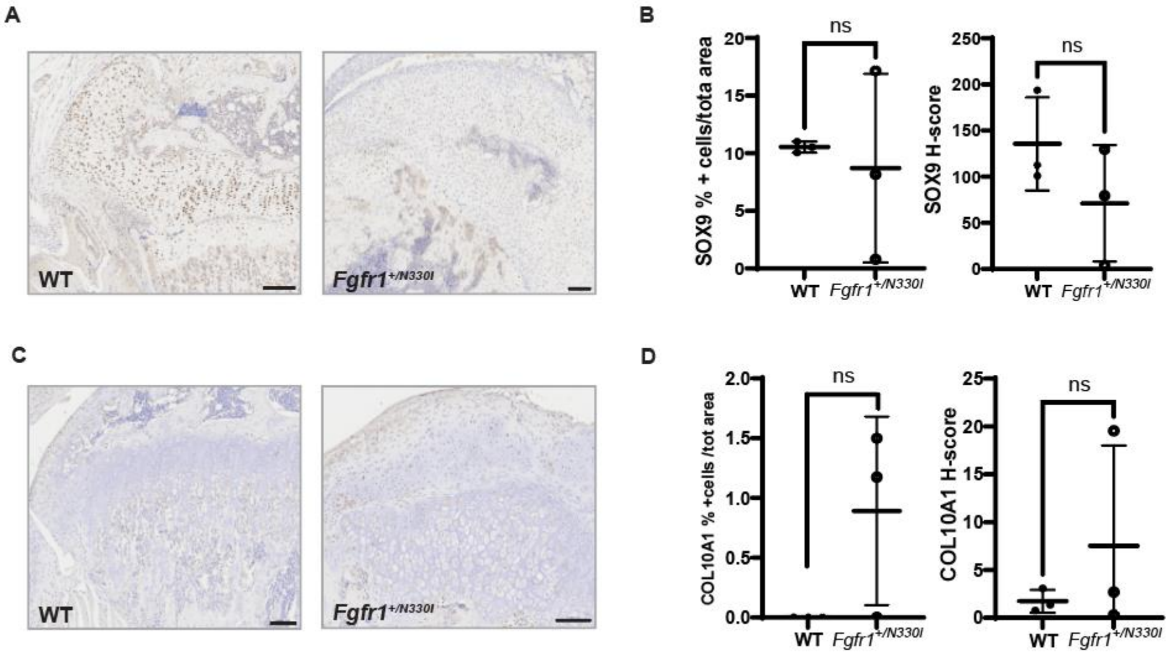

Supplemental Figure S2. Immunohistochemistry of SOX9 and Collagen X.

(A) Representative photomicrographs and **(B)** staining quantification showing similar protein levels of SOX9 in femurs derived from WT and *Fgfr1*$^{+/N330I}$ mice as shown by quantification of SOX9^{+} cells and relative H-score. **(C)** Representative photomicrographs and **(D)** staining quantification showing similar protein levels of Collagen X (COL10A1) in femurs of WT and *Fgfr1*$^{+/N330I}$ mice. Horizontal and vertical lines represent the mean ± SD of 3 mice/group (ns = not significant). Scale bar = 100 μm.

Supplemental Figure S3

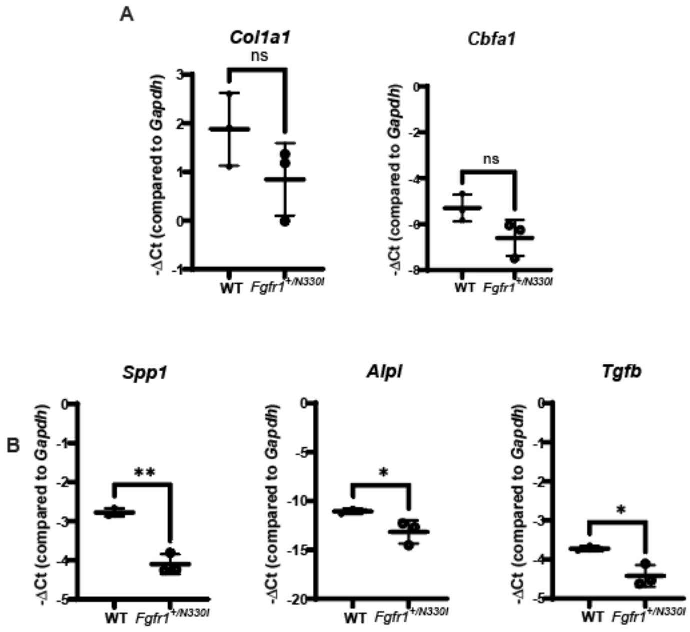

Supplemental Figure S3. *Fgfr1*$^{+/N330I}$ mice have reduced mRNA levels of osteoblast differentiation markers.

(A) WT and *Fgfr1*$^{+/N330I}$ mice show comparable messenger RNA (mRNA) expression of Collagen Type 1 (*Col1a1*) and Core-binding factor subunit alpha-1 (*Cbfa1*). **(B)** However, the mRNA levels of important osteogenic markers such as secreted phosphorylated protein 1 (*Spp1*), alkaline phosphatase (*Alpl*) and transforming growth factor beta (*Tgfb*) were significantly downregulated in the mutant mice. Horizontal and vertical lines represent the mean ± SD of 3 mice/group. (ns = not significant, * $p < 0.05$, ** $p < 0.01$).

Supplemental Table ST1

Gene name	Forward sequence	Reverse sequence
mCol1a1	5'- TGACTGGAAGAGCGGAGAGTA-3'	5'-AGACGGCTGAGTAGGGAACA-3'
mCbfa1/Runx2	5'-AGCCAGGTTCAACGATCTGA-3'	5'-GGACCGTCCACTGTCACTTT-3'
mSsp1	5'-GGCTGAATTCTGAGGGACTAACT-3'	5'-GCAATGCCAAACAGGCAAAAG-3'
mAlp1	5'-CAGGCCGCCTTCATAAGCA-3'	5'-CTCGTGGGGAGGGTCTGTTC-3'
mTgfb1	5'-ATTCCTGGCGTTACCTTGGT-3'	5'-GTGAGCGCTGAATCGAAAGC-3'
mGapdh	5'-GGCAAATTCAACGGCACA-3'	5'-GTTAGTGGGGTCTCGCTCCTG-3'

Supplemental Table ST1. Primers used for assessing gene expression in mouse cortical bone explants.

Supplemental Table ST2

MicroCT parameter	***Fgfr1***$^{+/N330I}$ **ossicles**	WT ossicles	p-value
Total volume (mm3)	31.6	13.1	0.004
BV/TV (%)	53.3	7.9	<0.0001
Tb.N (mm)	8.3	2.4	<0.0001
Tb.Th (mm)	0.09	0.06	<0.0001
Tb.Sp (mm)	0.13	0.45	0.0004
Conn.D (mm^3)	342.0	67.6	0.0001
BS/BV (mm)	23.2	51.0	p<0.0001
TMD ($mgHA/mm^3$)	690.8	772.3	0.002
DA (dimensionless)	1.09	1.32	0.001

Abbreviations: BV/TV, Bone volume/total volume; BS/BV, Bone surface/bone volume; Conn.D, Connectivity density; DA, Degree of anisotropy; TMD, Total mineral density; Tb.N, Trabecular number; Tb.Sp, Trabecular spacing; Tb.Th, Trabecular thickness.

Supplemental Table ST2. Microarchitectural parameters of in vivo ectopic ossicles (n = 6/cohort).

Supplemental Data SD1

AAACCCAGGAACTCCCCCTGCATGGCCCTCAAAGCCAGAGCAGAAAAGGGTACGCAGC
AGACGGGAGCCGTCCCTTTCTGCCTCTCCCCAGCCCTGTAACGGATGCGCTGCTCCCA
TGGCAAAAGGCAGGACCCGGAAAGGACCAGTGCACTGTATACCTTCCAGAACGGTCAA
CCATGCAGAGTGATGGGAGAGTCCGATAGAGTTACCCGCCAAGCACGTATACTCCCCCG
CATCCTCAAAGGAGACAATGCGTAGATGAAGCACCTCCATTTCCTTGTCGGTGGTATTAA
CTCCAGCAGTCTAGAAGAGACGACGGAAGCAAAATGGACAAGCCCGGGACATGAGACC
TCCGAGAGGCGGAGAAACGGAGGGGACAGAGGAGGGGACCAAAACCAGAAGAAGAAA
CGCTCCCCAGCATCTCCTTTGCAACAAGGCAAAAAAACCACACCACCAGCCAGCGAGA
CCTGTTAGAGGAAAACAGCGAGGCTCTGGAGGGGA

Supplemental Data SD1: Sequence of the homology-directed repair template used for generation of *Fgfr1*$^{+/N330I}$ mice.
